# Oxidative stress and the use of antioxidants in diabetes: Linking basic science to clinical practice

**DOI:** 10.1186/1475-2840-4-5

**Published:** 2005-04-29

**Authors:** Jeanette Schultz Johansen, Alex K Harris, David J Rychly, Adviye Ergul

**Affiliations:** 1 University of Tromso, Tromso, Norway; 2 University of Georgia College of Pharmacy, Athens, Georgia, USA; 3 Medical College of Georgia Vascular Biology Center, Augusta, Georgia, USA

**Keywords:** Antioxidants, Diabetes, Oxidative Stress

## Abstract

Cardiovascular complications, characterized by endothelial dysfunction and accelerated atherosclerosis, are the leading cause of morbidity and mortality associated with diabetes. There is growing evidence that excess generation of highly reactive free radicals, largely due to hyperglycemia, causes oxidative stress, which further exacerbates the development and progression of diabetes and its complications. Overproduction and/or insufficient removal of these free radicals result in vascular dysfunction, damage to cellular proteins, membrane lipids and nucleic acids. Despite overwhelming evidence on the damaging consequences of oxidative stress and its role in experimental diabetes, large scale clinical trials with classic antioxidants failed to demonstrate any benefit for diabetic patients. As our understanding of the mechanisms of free radical generation evolves, it is becoming clear that rather than merely scavenging reactive radicals, a more comprehensive approach aimed at preventing the generation of these reactive species as well as scavenging may prove more beneficial. Therefore, new strategies with classic as well as new antioxidants should be implemented in the treatment of diabetes.

## Introduction

It is a well-established fact that diabetes is a risk factor for cardiovascular disease [1,2]. While microvascular complications of diabetes include nephropathy and retinopathy, macrovascular complications resulting in atherosclerotic cardiovascular disease such as coronary artery disease, cerebrovascular disease and peripheral vascular disease are the leading cause of death in the diabetic population [3,4]. The Diabetes Control and Complications trial (DCCT) demonstrated that tight control of blood glucose is effective in reducing clinical complications significantly, but even optimal control of blood glucose could not prevent complications suggesting that alternative treatment strategies are needed [4]. Since numerous studies demonstrated that oxidative stress, mediated mainly by hyperglycemia-induced generation of free radicals, contributes to the development and progression of diabetes and related contributions, it became clear that ameliorating oxidative stress through treatment with antioxidants might be an effective strategy for reducing diabetic complications. To this end, several clinical trials investigated the effect of the antioxidant vitamin E on the prevention of diabetic complications. However, these trials failed to demonstrate relevant clinical benefits of this antioxidant on cardiovascular disease [5-7]. The negative results of the clinical trials with antioxidants prompted new studies focusing on the mechanisms of oxidative stress in diabetes in order to develop causal antioxidant therapy. In this article, sources of free radicals contributing to oxidative stress and the natural defense mechanisms in diabetes are briefly reviewed. Experimental and clinical evidence with respect to the use of conventional antioxidants in diabetes is summarized and causal therapy approaches with novel antioxidants are discussed.

### What is oxidative stress?

Oxidative stress is defined in general as excess formation and/or insufficient removal of highly reactive molecules such as reactive oxygen species (ROS) and reactive nitrogen species (RNS) [8,9]. ROS include free radicals such as superoxide (^•^O_2_^-^), hydroxyl (^•^OH), peroxyl (^•^RO_2_), hydroperoxyl (^•^HRO_2_^-^) as well as nonradical species such as hydrogen peroxide (H_2_O_2_) and hydrochlorous acid (HOCl) [8,10]. RNS include free radicals like nitric oxide (^•^NO) and nitrogen dioxide (^•^NO_2_^-^), as well as nonradicals such as peroxynitrite (ONOO^-^), nitrous oxide (HNO_2_) and alkyl peroxynitrates (RONOO) [8,10]. Of these reactive molecules, ^•^O_2_^-^, ^•^NO and ONOO^- ^are the most widely studied species and play important roles in the diabetic cardiovascular complications. Thus, these species will be discussed in more detail.

^•^NO is normally produced from L-arginine by endothelial nitric oxide synthase (eNOS) in the vasculature [8]. ^•^NO mediates endothelium-dependent vasorelaxation by its action on guanylate cyclase in vascular smooth muscle cells (VSMC), initiating a cascade that leads to vasorelaxation. ^•^NO also displays antiproliferative properties and inhibits platelet and leukocyte adhesion to vascular endothelium [8]. Therefore, ^•^NO is considered a vasculoprotective molecule. However, ^•^NO easily reacts with superoxide, generating the highly reactive molecule ONOO^-^, and triggering a cascade of harmful events as discussed below [8,11]. Therefore its chemical environment, i.e. presence of ^•^O_2_^-^, determines whether ^•^NO exerts protective or harmful effects.

**Figure 1:**
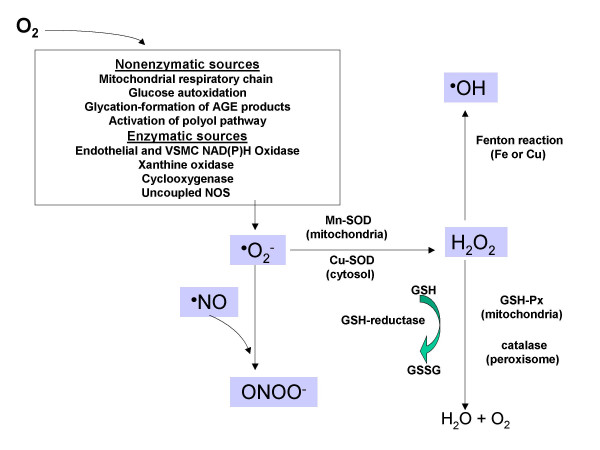
**Generation of reactive species in diabetes**. Highlighted in gray are some of the most important ROS and RNS in vascular cells. Oxygen is converted to ^•^O_2_^- ^via the activation of enzymatic and nonenzymatic pathways, which is then dismutated to H_2_O_2 _by SOD. H_2_O_2 _can be converted to H_2_O by catalase or glutathione peroxidase (GSH-Px) or to ^•^OH after reaction with Cu or Fe. Glutathione reductase regenerates glutathione (GSH). In addition, ^•^O_2_^- ^reacts rapidly with ^•^NO to form ONOO^-^.

Production of one ROS or RNS may lead to the production of others through radical chain reactions. As summarized in Fig. 1. ^•^O_2_^- ^is produced by one electron reduction of oxygen by several different oxidases including NAD(P)H oxidase, xanthine oxidase, cyclooxygenase and even eNOS under certain conditions as well as by the mitochondrial electron transport chain during the course of normal oxidative phosphorylation, which is essential for generating ATP [12-15]. Under normal conditions, ^•^O_2_^- ^is quickly eliminated by antioxidant defense mechanisms. ^•^O_2_^- ^is dismutated to H_2_O_2 _by manganese superoxide dismutase (Mn-SOD) in the mitochondria and by copper (Cu)-SOD in the cytosol [12]. H_2_O_2 _is converted to H_2_0 and O_2 _by glutathione peroxidase (GSH-Px) or catalase in the mitochondria and lysosomes, respectively. H_2_O_2 _can also be converted to the highly reactive ^•^OH radical in the presence of transition elements like iron and copper.

### Why are reactive species bad?

While ROS are generated under physiological conditions and are involved to some extent as signaling molecules and defense mechanisms as seen in phagocytosis, neutrophil function, and shear-stress induced vasorelaxation, excess generation in oxidative stress has pathological consequences including damage to proteins, lipids and DNA. These detrimental effects are briefly summarized in this section.

ROS can stimulate oxidation of low-density lipoprotein (LDL), and ox-LDL, which is not recognized by the LDL receptor, can be taken up by scavenger receptors in macrophages leading to foam cell formation and atherosclerotic plaques [16]. As will be discussed in greater detail in the next section, ^•^O_2_^- ^can activate several damaging pathways in diabetes including accelerated formation of advanced glycation end products (AGE), polyol pathway, hexosamine pathway and PKC, all of which have been proven to be involved in micro- and macrovascular complications. ^•^O_2_^- ^and H_2_O_2 _stimulate stress-related signaling mechanisms such as NF-κB, p38-MAPK and STAT-JAK resulting in VSMC migration and proliferation. In endothelial cells, H_2_O_2 _mediates apoptosis and pathological angiogenesis [15]. Furthermore, ^•^O_2_^- ^immediately reacts with ^•^NO generating cytotoxic ONOO^- ^and this reaction itself has several consequences. First, ONOO^- ^alters function of biomolecules by protein nitration as well as causing lipid peroxidation [8]. For example, potassium channels, which regulate the vasorelaxation response, are inhibited by nitration [17,18]. As recently reviewed by Turko et al, increased levels of nitrotyrosine are associated with apoptosis of myocytes, endothelial cells and fibroblasts in diabetes [8]. Second, ONOO^- ^causes single-strand DNA breakage which in turn activates nuclear enzyme poly(ADP-ribose) polymerase (PARP) [19]. Third, it decreases ^•^NO bioavailability causing impaired relaxation and inhibition of the antiproliferative effects of ^•^NO [9]. Furthermore, ONOO^- ^oxidizes tetrahydrobiopterin (BH_4_), an important cofactor for NOS, and causes uncoupling of NOS, which produces ^•^O_2_^- ^instead of ^•^NO [9]. ROS-induced peroxidation of membrane lipids alters the structure and the fluidity of biological membranes, which ultimately affects function [9,13-15]. All these pathological modifications contribute to the pathogenesis of vascular dysfunction.

### Sources of oxidative stress in diabetes

Direct evidence of oxidative stress in diabetes is based on studies that focused on the measurement of oxidative stress markers such as plasma and urinary F_2_-isoprostane as well as plasma and tissue levels of nitrotyrosine and ^•^O_2_^- ^[11,20-23]. There are multiple sources of oxidative stress in diabetes including nonenzymatic, enzymatic and mitochondrial pathways. Thus, we will first discuss these mechanisms and conclude with the recently proposed working plan for the initiation of oxidative stress and related vascular complications in diabetes.

Nonenzymatic sources of oxidative stress originate from the oxidative biochemistry of glucose. Hyperglycemia can directly cause increased ROS generation. Glucose can undergo autoxidation and generate ^•^OH radicals [8]. In addition, glucose reacts with proteins in a nonenzymatic manner leading to the development of Amadori products followed by formation of AGEs. ROS is generated at multiple steps during this process. In hyperglycemia, there is enhanced metabolism of glucose through the polyol (sorbitol) pathway, which also results in enhanced production of ^•^O_2_^-^.

Enzymatic sources of augmented generation of reactive species in diabetes include NOS, NAD(P)H oxidase and xanthine oxidase [21,22,24]. All isoforms of NOS require five cofactors/prosthetic groups such as flavin adenine dinucleotide (FAD), flavin mononucleotide (FMN), heme, BH_4 _and Ca^2+^-calmodulin. If NOS lacks its substrate L-arginine or one of its cofactors, NOS may produce ^•^O_2_^- ^instead of ^•^NO and this is referred to as the uncoupled state of NOS [9,21,22,24]. NAD(P)H oxidase is a membrane associated enzyme that consists of five subunits and is a major source of ^•^O_2_^- ^production [21,22,25,26]. Guzik et al. investigated ^•^O_2_^- ^levels in vascular specimens from diabetic patients and probed sources of ^•^O_2_^- ^using inhibitors of NOS, NAD(P)H oxidase, xanthine oxidase and mitochondrial electron transport chain. This study demonstrated that there is enhanced production of ^•^O_2_^- ^in diabetes and this is predominantly mediated by NAD(P)H oxidase. Furthermore, the NOS-mediated component is greater in patients with diabetes than in patients who do not have diabetes [22]. We have also observed that NAD(P)H oxidase activity is significantly higher in vascular tissue (saphenous vein and internal mammary artery) obtained from diabetic patients [27]. There is plausible evidence that PKC, which is stimulated in diabetes via multiple mechanisms, i.e. polyol pathway and Ang II, activates NAD(P)H oxidase [28].

**Figure 2:**
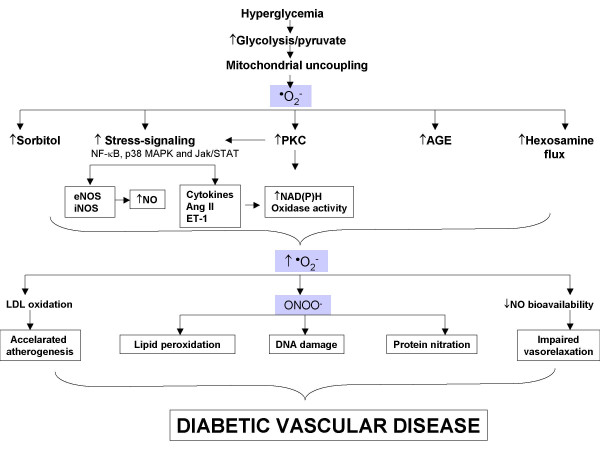
**Current working model for the generation of reactive species and downstream targets in diabetes. **Excess generation of mitochondrial ROS due to hyperglycemia initiates a vicious circle by activating stress-sensitive pathways such as NF-κB, p38 MAPK and Jak/STAT, polyol (sorbitol) and hexosamine pathways, PKC and AGEs. Enhanced production of AGEs, sorbitol and proinflammatory cytokines exerts a positive feedback on ROS and RNS synthesis and potentiates PKC-mediated vascular dysfunction by altering gene expression as well as vascular function and structure.

The mitochondrial respiratory chain is another source of nonenzymatic generation of reactive species. During the oxidative phosphorylation process, electrons are transferred from electron carriers NADH and FADH_2_, through four complexes in the inner mitochondrial membrane, to oxygen, generating ATP in the process [29]. Under normal conditions, ^•^O_2_^- ^is immediately eliminated by natural defense mechanisms. A recent study demonstrated that hyperglycemia-induced generation of ^•^O_2_^- ^at the mitochondrial level is the initial trigger of vicious cycle of oxidative stress in diabetes [30,31]. When endothelial cells are exposed to hyperglycemia at the levels relevant to clinical diabetes, there is increased generation of ROS and especially ^•^O_2_^-^, which precedes the activation of four major pathways involved in the development of diabetic complications. Nishikawa and colleagues elegantly demonstrated that generation of excess pyruvate via accelerated glycolysis under hyperglycemic conditions floods the mitochondria and causes ^•^O_2_^- ^generation at the level of Complex II in the respiratory chain. What is more important is that blockade of ^•^O_2_^- ^radicals by three different approaches using either a small molecule uncoupler of mitochondrial oxidative phosphorylation (CCCP), overexpression of uncoupling protein-1 (UCP1) or overexpression of Mn-SOD, prevented changes in NF-κB as well as polyol pathway, AGE formation and PKC activity. Based on this information, it has been postulated by several groups that mitochondrial ^•^O_2_^- ^is the initiating snowball that turns oxidative stress into an avalanche in diabetes by stimulating more ROS and RNS production via downstream activation of NF-κB-mediated cytokine production, PKC and NAD(P)H oxidase (Fig. 2). Thus, inhibition of intracellular free radical formation would provide a causal therapy approach in the prevention of oxidative stress and related vascular complications in diabetes.

### Natural defense against oxidative stress and antioxidants

Reactive species can be eliminated by a number of enzymatic and nonenzymatic antioxidant mechanisms. As discussed above, SOD immediately converts ^•^O_2_^- ^to H_2_O_2_, which is then detoxified to water either by catalase in the lysosomes or by glutathione peroxidase in the mitochondria (Fig. 1). Another enzyme that is important is glutathione reductase, which regenerates glutathione that is used as a hydrogen donor by glutathione peroxidase during the elimination of H_2_O_2_. Maritim and colleagues recently reviewed in detail that diabetes has multiple effects on the protein levels and activity of these enzymes, which further augment oxidative stress by causing a suppressed defense response [9]. For example, in the heart, which is an important target in diabetes and prone to diabetic cardiomyopathy leading to chronic heart failure, SOD and glutathione peroxidase expression as well as activity are decreased whereas catalase is increased in experimental models of diabetes [9,32,33]. In patients with chronic heart failure, all three enzymes are decreased in the smooth muscle [34] and exercise training can upregulate the expression and activity of antioxidant enzymes. Increased isoprostane levels in diabetic patients with chronic heart failure are correlated with antioxidant status and disease severity [35]. Thus, modulation of these enzymes in target organs prone to diabetic complications such as heart and kidney may prove beneficial in the prevention and management of heart failure and kidney failure.

Nonenzymatic antioxidants include vitamins A, C and E; glutathione; α-lipoic acid; carotenoids; trace elements like copper, zinc and selenium; coenzyme Q_10 _(CoQ_10_); and cofactors like folic acid, uric acid, albumin, and vitamins B_1_, B_2_, B_6 _and B_12_. Alterations in the antioxidant defense system in diabetes have recently been reviewed [11]. Glutathione (GSH) acts as a direct scavenger as well as a co-substrate for GSH peroxidase. It is a major intracellular redox tampon system. Vitamin E is a fat-soluble vitamin that prevents lipid peroxidation. It exists in 8 different forms, of which α-tocopherol is the most active form in humans. Hydroxyl radical reacts with tocopherol forming a stabilized phenolic radical which is reduced back to the phenol by ascorbate and NAD(P)H dependent reductase enzymes [36,37]. CoQ_10 _is an endogenously synthesized compound that acts as an electron carrier in the Complex II of the mitochondrial electron transport chain. Brownlee et al reported that this is the site of ^•^O_2_^- ^generation under hyperglycemic conditions [30,31]. CoQ_10 _is a lipid soluble antioxidant, and in higher concentrations, it scavenges ^•^O_2_^- ^and improves endothelial dysfunction in diabetes [38-40]. Vitamin C (ascorbic acid) increases NO production in endothelial cells by stabilizing NOS cofactor BH_4 _[41]. α-Lipoic acid is a hydrophilic antioxidant and can therefore exert beneficial effects in both aqueous and lipid environments. α-lipoic acid is reduced to another active compound dihydrolipoate. Dihydrolipoate is able to regenerate other antioxidants such as vitamin C, vitamin E and reduced glutathione through redox cycling [41]. Thus, both experimental and clinical studies summarized in the next sections utilized these naturally occurring antioxidants, especially vitamins C, E and α-lipoic acid, in order to delineate the role of oxidative stress in the development of vascular complications of diabetes.

### Evidence from experimental models

A multitude of *in vivo *studies have been performed utilizing antioxidants in experimental diabetic models. The effects of antioxidants on oxidative stress are measured through certain observable biomarkers. These markers include the enzymatic activities of catalase, SOD, GSH-Px, and GSH-reductase, as well as thiobarbituric acid reactants (TBARS) levels, an indirect measurement of free-radical production that has been shown to be consistently elevated in diabetes. Normalization of the activity levels of any of these markers, and ultimately, the balance of free-radical production/removal, would be an effective method to reduce ROS-induced damage. Many animal studies have been completed with this aim in mind and indeed have shown that diabetes-induced alterations of oxidative stress indicators can be reversed when the animals are treated with various antioxidants. It should be noted that a plethora of studies have been done with numerous antioxidant compounds. We will, however, only cover a select few within the scope of this review, specifically the compounds for which a corresponding human clinical trial has been conducted

Mekinova et al. demonstrated that supplementation of streptozotocin (STZ) diabetic rats with vitamins C, E, and beta-carotene for 8 weeks produced a significant reduction of TBARS levels, GHS, and GHS-Px, an increase in Cu-SOD, and no change in catalase activity in kidneys [42]. Treatment with vitamins C and E was also shown to decrease urinary albumin excretion, glomerular basement membrane thickness, and kidney weight in STZ diabetic rats [43]. In the same study, vitamins C and E significantly lowered malondialdehyde (TBARS) levels and GSH-Px activity while increasing catalase and SOD activities when compared to unsupplemented diabetic animals [43]. A study by Cinar et el. demonstrated that supplementation with vitamin E significantly lowered liver and lung TBARS levels and improved impaired endothelium-dependent vasorelaxation in STZ diabetic rat aorta [44].

α-lipoic acid, which is involved in mitochondrial dehydrogenase reactions, has gained a considerable amount of attention as an antioxidant. Studies have demonstrated that intraperitoneal administration of α-lipoic acid to STZ diabetic Wistar rats normalizes TBARS level in plasma, retina, liver, and pancreas [45]. In the same study, Obrosova et.al observed a reduction of GSH activity in diabetic retina and that supplementation with α-lipoic acid produced no change [45]. However, another study demonstrated an increase in aortic GSH-Px in STZ diabetic rats that was normalized by treatment with α-lipoic acid [46]. Additionally, increased maximum contractile responses in diabetic aortic rings were ameliorated with α-lipoic acid treatment [46].

SOD activity is undoubtedly important to the regulation of oxidative status in diabetes. However, there is variation as to the status of this enzyme in the diabetic state. Some studies have reported decreased SOD activity [43,45] while others have shown increases [47] or no change in the enzyme [42,48]. α-lipoic acid has been observed to normalize diabetes-induced decreases of SOD in rat heart [48] and retina [45]. One study demonstrated that treatment of STZ diabetic rats with α-lipoic acid reverses SOD-induced vasorelaxation, potentially due to the elimination of excess superoxide/hydrogen peroxide and the recovery of basal NO [46]. A recent study by Brands et al. investigated the effect of oxidative stress in the development of hypertension in diabetes using the SOD mimetic tempol in a Type 1 model of diabetes where NOS is pharmacologically inhibited with a NOS inhibitor, L-NAME [49]. In this model, hyperglycemia causes hypertension implicating an important role for NO. Results of this study showed that if ^•^O_2_^- ^is eliminated by tempol early in the disease process, the hypertension and decrease in glomerular filtration precipitated by diabetes are prevented.

In summary, there are differences in response to antioxidants in experimental diabetes in the prevention of cardiovascular complications. Studies in experimental models provide a foundation for the clinical studies but results should be interpreted cautiously since the experimental models of diabetes, duration and type of antioxidant treatment and markers of oxidative stress investigated in these studies exhibit a wide range.

### Evidence from clinical trials

Although studies with antioxidants in experimental models as well as observational studies strongly suggest that antioxidants should confer beneficial effects in reducing cardiovascular complications in diabetes, clinical evidence for the use of antioxidants is not solid. It should be emphasized that clinical trials with antioxidants in diabetes are limited and majority of these trials focused on the use of vitamin E and C and lately α-lipoic acid. Thus, we will attempt to group the clinical trials by the antioxidants used.

Small trials with vitamin E demonstrated beneficial cardiovascular effects. In a double-blind, placebo-controlled, randomized study, vitamin E supplementation (1000 IU/day) for three months in patients with Type 1 diabetes (n = 41) significantly improved endothelium-dependent vasorelaxation [50]. In another study, Beckman et al. reported that administration of vitamin E (800 IU/day) and C (1000 mg/day) combination for six months had a positive effect on endothelium-dependent vasorelaxation in Type 1 diabetic patients (n = 26) but had no effect in Type 2 diabetes (n = 23) [51]. Gaede et al reported that vitamin E (680 mg/day) and C (1250 mg/day) combination significantly improved renal function in Type 2 diabetes [52].

Other clinical trials on a larger scale include the Heart Outcomes Prevention Evaluation (HOPE) trial [53], Secondary Prevention with Antioxidants of Cardiovascular Disease in End Stage Renal Disease (SPACE) trial [54], the Steno trial [55], the Primary Prevention Project (PPP) trial [56] and the Study to Evaluate Carotid Ultrasound Changes in Patients Treated With Ramipril and Vitamin E (SECURE) trial [57].

The HOPE trial enrolled patients 55 years of age or older who were at high risk for cardiovascular disease and recruited significant number of patients with diabetes. This study had a 2 × 2 factorial design where in one arm patients were randomized to vitamin E (400 IU/day) or placebo and in the other arm of the study patients were randomized to ramipril (10 mg/day) or placebo [53]. Results with vitamin E and ramipril were evaluated separately as compared to respective placebo groups. In the vitamin E arm, 4761 patients received vitamin E and 4780 patients were given placebo. In the treatment and placebo groups, the number of patients with diabetes was 1838 and 1816, respectively. The primary endpoint was a composite of myocardial infarction, stroke and death from cardiovascular causes. The trial was stopped for ethical reasons after 4.5 years follow-up by the recommendations of an independent data and safety monitoring board based on the beneficial effects of ramipril on cardiovascular events in the concurrent treatment group and lack of effect in the vitamin E treatment group. Results of the study were published in 2000 and demonstrated that there was no significant difference in the primary outcome between vitamin E and placebo groups [53]. Analyses of the secondary endpoints of the study, which included total mortality, hospitalizations for heart failure and unstable angina, revascularization and nephropathy, were recently published [58], and again vitamin E supplementation for 4.5 years failed to provide any benefit in cardiovascular outcomes or nephropathy. It was also reported that there were no significant adverse events associated with vitamin E. The HOPE trial was the largest trial conducted thus far for the use of antioxidants in diabetes. The SECURE trial was designed as a substudy of the HOPE trial to evaluate the effects of long-term treatment with ramipril and vitamin E on atherosclerosis progression in high-risk patients. In this trial, 732 patients who had vascular disease or diabetes were randomized to two doses of (2.5 or 10 mg/d) ramipril and vitamin E (400 IU/day) or placebo and progression of atherosclerosis was monitored by B-mode carotid ultrasound. While ramipril slowed down atherosclerotic changes, vitamin E had no effect as compared to placebo group.

The SPACE trial recruited 196 hemodialysis patients with preexisting cardiovascular disease who were assigned to either placebo (n = 99) or 800 IU/day vitamin E (n = 97) for 2 years. 43% of the patients in each group had diabetes. The primary endpoint was a composite of myocardial infarction, stroke, peripheral arterial disease or unstable angina. There was a 46% decrease in the primary end point events in the vitamin E group and this was mainly due to a 70% reduction in total myocardial infarction [54]. The PPP trial was a randomized trial again with a 2 × 2 design to evaluate the effect of low dose aspirin (100 mg/day) and vitamin E (300 mg/day) on the prevention of cardiovascular complications in high-risk patients. Similar to the studies discussed above, the primary endpoint was a composite of cardiovascular death, stroke or myocardial infarction. Out of the 4784 patients recruited, 1031 had diabetes. The PPP trial was stopped prematurely by the recommendations of an independent data and safety monitoring board based on the consistent beneficial effects of aspirin as compared to placebo group. However, there was no significant effect of vitamin E treatment either in diabetic or nondiabetic subjects. Lastly, the Steno-2 trial compared the effect of a multifactorial intensive therapy (n = 80) with that of conventional treatment (n = 80) on modifiable risk factors for cardiovascular disease in patients with Type 2 diabetes [55]. In the intensive treatment group, patients received pharmacotherapy that targeted hyperglycemia, dyslipidemia, hypertension and microalbuminuria including daily supplementation of vitamin C (250 mg), E (100 mg), folic acid (400 mg) and chromium picolinate (100 mg) as well as behavior modification including low-fat diet, exercise and smoking cessation. The control group received conventional therapy as recommended in national guidelines. The intensive therapy resulted in almost a 50% decrease in the risk of cardiovascular events providing evidence that a multifactorial approach is superior to conventional therapy for the prevention of oxidative stress-induced vascular complications in diabetes.

Studies with α-lipoic acid are approved for the treatment of diabetic neuropathy and results are more promising than those obtained with vitamin E. In the Alpha Lipoic Acid in Diabetic Neuropathy (ALADIN) study, infusion of α-lipoic acid (>600 mg) significantly improved patient symptoms [59]. The ALADIN II Study demonstrated that long-term (24 months) use of α-lipoic acid (600 or 1200 mg) improved nerve function [60]. ALADIN III, a randomized multicenter double-blind placebo controlled study, showed that in a cohort of 509 patients, 600 mg α-lipoic acid administration for 6 months improved neuropathy impairment score as early as 19 days, which was maintained up to 7 months [61]. The DEKAN (Deutsche kardiale autonome neuropathie) study evaluated the effect of 800 mg α-lipoic acid or placebo in diabetic patients with cardiac autonomic neuropathy for 4 months and showed that heart rate variability, an indicator of cardiac autonomic neuropathy, significantly improved with α-lipoic acid treatment [62]. The SYDNEY trial investigated the effect of α-lipoic acid treatment on sensory symptoms of diabetic polyneuropathy as assessed by the Total Symptom Score. Administration of this antioxidant over a 3-week period improved sensory symptoms such as pain, prickling and numbness [63]. A recent meta-analysis of trials with α-lipoic acid concluded that treatment with intravenous α-lipoic acid (600 mg/day) over a 3-week period is safe and effective in improving positive neuropathic symptoms as well as neuropathic deficits [64].

In summary, clinical trials with conventional antioxidants in diabetic patients are limited. For major cardiovascular outcomes, vitamin E failed to provide any benefit. However, when study population was limited to diabetic patients alone as done in diabetic neuropathy trials, α-lipoic acid has proven to be effective. As further discussed under Perspectives, this antioxidant may be a viable option in trials focusing on cardiovascular outcomes in diabetes.

In addition to the many antioxidants examined above, a number of commonly used drugs have shown promising antioxidant activity in addition to their primary pharmacological activity. These drugs include thiazolidinediones (TZDs), HMG-CoA reductase inhibitors (statins), and inhibitors of the renin-angiotensin system.

Thiazolidinediones (TZDs) have been shown in many animal studies to have antioxidant effect. In one study, pioglitazone-treated rats had reduced urinary excretion of isoprostane, a marker of oxidative stress [65]. In a trial with type-2 diabetic rats, Bagi et al demonstrated that treatment with rosiglitazone reduced NAD(P)H-derived ROS and increased the activity of catalase [66]. Another study using type-2 diabetic rats found that treatment with troglitazone lowered hydroperoxides and decreased SOD activity [67]. A study using troglitazone and pioglitazone in type-2 diabetic rats found that both agents reduced TBARS levels and increased the aortic vasorelaxation response [68].

There is substantial evidence from in vitro studies that statins exert an antioxidant effect. Studies have demonstrated that statin therapy markedly reduces oxidative stress markers (such as nitrated tyrosine) in animals [69]. Although the mechanisms for these actions are still being elucidated, Takayama et al have demonstrated in canine models that the antioxidant effect of statins is at least partially due to inhibition of NAD(P)H oxidase [70]. Statins have also been shown to stimulate the activity of the antioxidant enzyme thioredoxin [71]. Additionally, statin therapy has been shown to stimulate the activity of paraoxonase (PON), which has a putative role in protecting LDL from oxidation [72]. Oxidation of LDL ex vivo has been shown to be inhibited by long-term statin therapy, an effect thought to be partly due to the binding of the statins to the LDL itself. It seems likely from the above studies that the antioxidant actions of statins are manifested via a variety of mechanisms.

Inhibitors of Angiotensin II (Ang II) activity, such as Angiotensin Converting Enzyme Inhibitors (ACEIs) and Angiotensin II receptor blockers (ARBs) have shown some beneficial effects that may stem from their antioxidant properties. Angiotensin II has been shown to increase ROS levels in animal studies, through stimulation of NAD(P)H oxidase activity [15,73]. Studies have suggested that this effect also occurs in humans [73,74]. Ang II has also been implicated in upregulating the expression of the LOX-1 receptor, which is specific for oxidized LDL cholesterol. Inhibition of the generation of Ang II, whether by ACEI or ARB, should therefore attenuate these deleterious processes. Indeed, Berry et al have shown that treatment with ACEI or ARB decreases ^•^O_2_^- ^levels in the human vasculature [75].

In summary, many of the agents which are a mainstay of pharmacotherapy in diabetes have been shown to have antioxidant properties in addition to their primary pharmacological actions. These antioxidant properties may be a contributing factor to the therapeutic efficacy of these agents. Their antioxidant properties make the case for use of these drugs even more compelling. Particularly in light of the lackluster results seen in clinical trials with antioxidant supplementation, health care providers should redouble their efforts to ensure adequate usage of the demonstrably effective agents summarized above.

### *Perspectives*- Is there a role for antioxidant treatment in diabetes?

Although the clinical trials conducted to date failed to provide adequate support for the use of antioxidants in diabetes, it is still to early to reach a definitive conclusion on this issue. As discussed above, with the exception of alpha-lipoic acid studies in diabetic neuropathy, data from clinical trials are limited. The majority of studies were not designed to assess the effect of antioxidant use specifically in diabetic patients. This is an important point because diabetic patients represent a population in whom oxidative stress is much higher than in the general population. As was seen in the SPACE trial of patients on hemodialysis, patients exposed to very high oxidative stress responded favorably to vitamin E supplementation [54]. It is possible that antioxidants would be more demonstrably effective in a patient population chosen on the basis of elevated levels of oxidative stress. Unfortunately, none of the studies to date effectively assessed the baseline oxidative stress of the enrolled patients using any of the commonly accepted markers of inflammation.

The human trials to date used endpoints that were not directly related to oxidative stress, but rather gross markers of overall cardiovascular health, such as effect on mortality. The studies failed to assess the duration of the diabetic disease states, arguably a large confounding variable. In assessing oxidative stress and the effects of antioxidants thereon, specific markers of oxidative stress should be measured.

With respect to the specific antioxidants studied, their selection was based on epidemiological and observational data, and in the absence of any solid grasp of the underlying mechanisms of action. Whereas observational studies are based on whole populations and reflect the lifelong influence of dietary habits, most of the studies were five years duration or less and included older patients (average age 65.4 years). It is possible that the study populations represented patients in whom the disease states had progressed too far to be amenable to antioxidant intervention.

In all likelihood, the choice and dose of antioxidant might be very important. The clinical trials focused mainly on the use of vitamin E. Negative results with vitamins cannot be generalized to all antioxidants. As has been eloquently argued elsewhere, treating the antioxidant vitamins as a single class of compounds with expected similar effects inappropriately disregards their wide range of chemical properties and pharmacodynamics [76]. Clinical trials to date have been conducted without any real understanding of the mechanisms of action or the concentrations of the various agents seen at different physiological sites. Indeed, there is not sufficient evidence to demonstrate that vitamin E reaches target cells.

Recently, it has been postulated that antioxidant potency of vitamins such as C and E is limited because these antioxidants work as scavengers of existing excess reactive species in a stoichiometric manner and this approach represents a symptomatic approach to oxidative stress-associated clinical problems [77]. Based on the new developments in our understanding of the pathophysiology of oxidative stress, it is clear that strategies to block the formation of reactive radicals will provide a targeted and causal approach to provide conclusive evidence whether antioxidants should be part of the cardiovascular treatment plan in diabetes. Candidate agents include low molecular weight mitochondrial and cytosolic SOD and catalase mimetics, L-propionyl carnitine, PKC-β inhibitor LY-333531, peroxynitrite catalyst FP15 and mitochondrial uncoupler DNP [9,77,78].

Given the number of shortcomings in the clinical trials, it seems clear that more research on the use of antioxidants in the prevention of cardiovascular complications in diabetes is necessary and strongly encouraged. From a clinical viewpoint, however, efforts for the prevention of diabetic complications should seek to maximize the benefits of proven therapeutic strategies including appropriate life style changes and controlling blood pressure, blood glucose and lipids.

In conclusion, the amount of evidence on the harmful effects of oxidative stress on vascular function and the link to pathophysiological mechanisms underlying diabetic complications is compelling. While the lack of clinical evidence on the beneficial effects of antioxidant vitamins in diabetes management should not deter us from more basic and clinical research on this issue, practice guidelines that are based on the results of numerous clinical trials should be our guide to evidence-based medicine in the prevention of cardiovascular disease in diabetes. The recent American Heart Association science advisory on the subject of antioxidant vitamins and cardiovascular disease asserted that there is insufficient evidence to justify the use of antioxidant vitamins for cardiovascular disease risk reduction [79]. Hopefully, further research into the pathophysiology of oxidative stress and the role of antioxidant therapy will lead to appropriately-designed clinical trials in which the promise of antioxidant therapy will be realized.

## Competing Interests

The author(s) declare that they have no competing interests.

## Authors' Contributions

AKH and JSJ contribute equally to writing the evidence-based sections and drafting of this review. DR was responsible for critical revision and formatting. AE participated in all aspects and areas of this review.
